# On Ganglion, Simple and Compound

**Published:** 1898-04-23

**Authors:** E. Percy Paton

**Affiliations:** Surgical Registrar to Westminster Hospital, &c.


### Medical Progress and Hospital Clinics.

[*The Editor will be glad to receive offers of co-operation and contributions from members of the profession. All letters should be addressed to* The Editor, at the Office, 28 & 29, Southampton Street, Strand, London, W.C.

### ON GANGLION, SIMPLE AND COMPOUND.

By E. Percy Paton, M.D., M.S., F.R.C.S., Surgical Registrar to Westminster Hospital, &c.

(*Concluded from page 42.*)

The compound ganglion is a much more serious trouble than the simple, but, fortunately, is much less common.

It is practically only found affecting the complicated synovial sheaths of the palm of the hand, and commencing here extends up under the anterior annular ligament on to the flexor aspect of the wrist.

As it commonly does not appear to give much trouble in its early stages, it is usually first seen as a smooth, elastic, or soft, fluctuating swelling just above the annular ligament, with a convex upper margin, or it may extend for some little distance up the flexor aspect of the arm. Frequently there is no obvious swelling in the palm of the hand, the resistance of the palmar fascia preventing this; but on making firm pressure on the swelling above the wrist there is a sensation of fluid being forced into the palm, and so the structures here rising can usually easily be made out; at the same time a sort of soft, grating sensation may often be detected, due to the presence of loose bodies ("melon seed bodies"), or to the enlarged synovial fringes rubbing over one another. In some cases the swelling extends along the flexor aspect of the thumb and little finger, with which the general palmar sheaths may communicate, the thumb being more commonly affected than the little finger. A compound palmar ganglion is not infrequently associated with disease, usually of a tuberculous nature, of the wrist-joint, and in every case this joint should be carefully examined, to exclude any such trouble which, if present, would naturally be the more serious affection, the ganglion being merely a complication.

Wrist-joint disease will be evidenced by the usual signs of pain, especially on movement, which is more limited than normal, swelling on the dorsal aspect as well as on the palm, and when the disease is advanced grating in the joint and perhaps evidence of suppuration. The walls of a compound ganglion are composed of the thickened synovial sheaths, which, if the case owes its origin to tubercle, will present the usual appearances typical of tuberculous granulation tissue, such as is found in tuberculous disease of the synovial membrane of the knee.

In simple cases, not due to tubercle, the fringes which are normally present in the synovial membrane are often greatly magnified in size, and frequently are larger at their extremities than their bases. These masses may then become detached, and form the melon-seed bodies which are sometimes found in very large numbers in the ganglion. These bodies may also be formed by aggregations of inflammatory lymph, and probably also in other ways, their smoothed surface being due to their friction against each other. The prognosis of compound ganglion depends largely on its cause as well as its treatment. Should it be tuberculous, and of course still more so if secondary to wrist-joint trouble, it need hardly be said that it is most unsatisfactory, and, indeed, in the latter case, will not improbably lead to loss of the hand; but in simple cases a satisfactory result from careful treatment may be anticipated.

Treatment.—It is most important, whatever line of treatment is adopted, to ensure by strict asepsis that no suppuration takes place, as, should this occur, very great damage will result, as the septic inflammation will track rapidly in all directions in the palm, and up the tendon sheaths of the arm, and even should no sloughing of tendons result, when healing has taken place, there will be so much matting of these structures to one another, to their sheaths, and to the surrounding parts that the utility of the hand will be very much marred, owing to the greatly reduced mobility of the fingers. The simplest procedure is aseptic drainage, which is done as follows: The hand and arm being rendered surgically clean, an incision is made into the swelling just above the anterior annular ligament of the wrist, and parallel to the course of the tendons, care being taken to avoid the median nerve, which is here the most superficial structure, lying immediately under and, more or less, in the distended synovial sheath. A long-eyed probe is then passed under the ligament into the palm, and cut down upon, the superficial palmar arch, the surface marking for which is a line found by abducting the thumb and then drawing a line along its ulnar border across the palm, is avoided by cutting down on the probe nearer the fingers than this landmark. The probe is now brought out through the opening made, and after squeezing out all the contents of the ganglion a piece of drainage tube, or several strands of horse-hair or silkworm gut, are drawn through from one opening to the other. The hand is then dressed with the usual antiseptic dressings, fixed on a short dorsal splint, and put in a sling, being left untouched for about ten days. The drainage is then removed, and the wounds allowed to close. Should this not succeed it may be repeated; but before leaving in the drainage, which should be left in a somewhat longer time, the sheaths may be thoroughly irrigated with a strong antiseptic, such as 1-40 carbolic or strong iodine lotion. In the event of failure again resulting, a more extensive procedure must be undertaken, the upper incision being prolonged right across the annular ligament into the palm; and, indeed, in cases where there is much thickening of the sheath, this may be done at first, and should always be done in tuberculous cases. By this incision a very free exposure is obtained, and by careful dissection the thickened synovial membrane should, as far as possible, be cut away, having due regard, of course, to the nerves and arteries in the vicinity. In tuberculous cases a little iodoform may be sprinkled over the wound. The divided annular ligament is then united with silk sutures, and the rest of the wound closed except for a small portion at either end. The hand is placed on a splint and the dressings left undisturbed, unless there is some good reason for doing otherwise, for ten days. Subsequently, when the wound is healed, and unless the case be tuberculous, when longer rest is required, passive movement of the fingers, combined with massage, will greatly overcome any stiffness which at first results.

Should suppuration by any misfortune occur, the freest possible drainage must at once be instituted, the parts thoroughly irrigated with some strong antiseptic, and boracic fomentations, or, better still, an arm bath started so that speedy subsidence of the inflammation may be obtained. Should these measures not succeed, and should extensive destruction and sloughing take place in the palm, with tracking up the arm, amputation will be the only resort; and even under the most favourable circumstances much loss of movement will be almost sure to result when the suppuration has ceased and the wounds have closed. In cases in which the wrist-joint is the primary trouble, it is the disease here which requires treatment; and in all except very slight cases, when the general synovial sheath on the hand has shared in the disease, very extensive operative interference will be necessary, and amputation through the fore-arm will be a very probable termination of the case.

